# Neutralisation techniques used by defendants charged with animal welfare offences in Finland

**DOI:** 10.1017/awf.2024.32

**Published:** 2024-09-16

**Authors:** Elli Valtonen, Laura Hänninen, Anna Valros, Tarja Koskela

**Affiliations:** 1 Faculty of Veterinary Medicine, University of Helsinki, Helsinki, Finland; 2 Law School, University of Eastern Finland, Joensuu, Finland

**Keywords:** animal hoarding, animal welfare, companion animals, neutralisations techniques, production animals, violence against animals

## Abstract

Animal welfare offences encompass a heterogeneous range of crimes, including violence and various forms of negligence toward animals’ needs. However, there is limited understanding of the offenders’ rationalisations concerning their criminal behaviour against animals, despite this information being essential for enhancing the prevention of these crimes. Our data comprised 1,443 judgements in animal welfare offences in Finland between January 2011 and May 2021. We categorised the rationalisations used by defendants and identified differences between offender profiles according to the offence type. Nearly all defendants responded to the charges. Overall, defendants appealed most often to their challenging circumstances, e.g. a lack of resources. Defendants charged with offences against production animals offered more explanations than the other defendants and often denied their responsibility for the animals, or having caused them any harm, and appealed to financial problems, weather conditions, and having too many animals. Moreover, they frequently challenged the norms, appealing in particular to the immorality of the authorities, who were mostly official veterinarians. Defendants charged with animal hoarding offences rationalised their actions similarly to animal farmers, whereas those charged with violent crimes against animals more often cited provocative or otherwise problematic behaviour of the animal victim. Our results support the observation that farmers may perceive official animal welfare supervision negatively. Violent animal welfare crimes and animal hoarding stand out as distinctive types of crime at the level of rationalisations. The differences between offence types and offenders’ underlying motivations should be considered when developing animal welfare control, agricultural support systems, and crime prevention.

## Introduction

Animal welfare offences encompass a heterogenous range of crimes, including violence and various forms of negligence toward animals’ physiological and behavioural needs (Maher *et al.* 2017; van Wijk *et al.* 2018; Valtonen *et al.* 2023). It has been shown that the offender profile varies between different forms of crime against companion animals: violent offenders are often young urban men (Arluke & Luke 1997; van Wijk *et al.* 2018; Valtonen *et al.* 2023) whereas animal hoarding behaviour is more common among middle-aged and elderly women (Patronek 1999; Paloski *et al.* 2017). However, we know less about the profiles of those offenders who commit crimes against production animals. In Australia, the average penalties for crimes against production animals were harsher and the number of animal victims higher when compared to crimes against companion animals (Morton *et al.* 2018), which may indicate differences in the severity of the offences and/or in the argumentation lines of the defence.

In Finland, animal welfare offences can be convicted as animal welfare infringement (Animal Welfare Act 1996), petty, basic, or aggravated animal welfare offences (Criminal Code of Finland 1889; CCF). Animal welfare infringement and petty offences are punishable with a fine, basic animal welfare offences with a fine or imprisonment, and aggravated animal welfare offences with imprisonment. In addition, a permanent or temporary ban on the keeping of animals can be imposed as a precautionary measure due to an offence regardless of the penalty. The court may order animals subjected to a crime and/or owned by the offender to be forfeited to the state (CCF 1889).

A large body of research shows that criminal offenders utilise various techniques to rationalise their actions. According to the neutralisation theory introduced by Sykes and Matza (1957), criminal behaviour does not necessarily indicate ignorance or indifference towards social norms. Instead, preceding their action, ‘neutralisation techniques’ allow the delinquent individual to explain how they do not violate a norm, or how they cannot choose an alternative course of action. Sykes and Matza established their model of five classic neutralisation techniques that offenders use to justify their criminal behaviour in advance of these actions: denial of responsibility; denial of injury; denial of the victim; condemnation of condemners; and the appeal to higher loyalties. Scott and Lyman (1968) suggested some new categories and divided these techniques into justifications and excuses, which are mainly presented after the criminal action. According to them, justifications are offered when the actor accepts responsibility for their action but rationalises their reasons for committing it, whereas with excuses they seek to mitigate their responsibility, fully or partly, for the action or its consequences. Goffman (1971) introduced the concept of ‘remedial work’, which includes accounts and apologies. Subsequently, the neutralisation theory has been well established in research, and the techniques have been categorised in various ways (e.g. Coleman 1994; Cromwell & Thurman 2003; Kaptein & van Helvoort 2019) and studied in different contexts of criminal and other antisocial behaviour, such as domestic (Cavanagh *et al.* 2001), medicalised (Johnston & Kilty 2016), and gun violence (Pogrebin *et al.* 2006), shoplifting (Cromwell & Thurman 2003), and police crimes (Gottschalk 2012).

Acceptance of neutralisations has been shown to precede criminal behaviour (e.g. Agnew 1994; Morris & Copes 2012). However, as Presser (2004), Maruna and Copes (2005), and Ugelvik (2012) point out, the relationship between rationalisations and offending does not appear to be causal but rather a dynamic process of mitigating guilt, shame, or cognitive dissonance, and maintaining self-esteem and identity. Further, it has been shown that the explanatory style of shifting the responsibility to sources less central to the perpetrator’s personality also has a positive effect on other people’s perceptions of the wrongdoer (Snyder & Higgins 1988), and that offering excuses and expressing remorse has a positive effect on the evaluation of the perpetrator (Cornell *et al.* 2009), even when sentencing for serious crimes (Kleinke *et al.* 1992).

Neutralisation theory has also been applied to crimes against animals. For example, ‘dogmen’ who arrange dog fights utilised several neutralisation techniques: denial of injury, condemnation of the condemners, appeal to higher loyalties, and a defence that characterises dogmen as ‘good people’ (Forsyth & Evans 1998). According to media articles, animal hoarders offered various excuses and justifications, from denying their responsibility to being a Good Samaritan or a victim of the system (Vaca-Guzman & Arluke 2005). Farmers who were accused of neglect claimed that they had been mistreated by the authorities and suffered from financial problems (Andrade & Anneberg 2014; Devitt *et al.* 2015; Väärikkälä *et al.* 2020), or health problems, and complained that they had too many animals (Väärikkälä *et al.* 2020). Motives for violent animal abuse were studied by interviewing undergraduate students (Newberry 2018), and inmates (Hensley & Tallichet 2005), and by examining offenders’ explanations in police records (van Wijk *et al.* 2018), with offenders’ motives appearing to include, e.g. by anger, amusement, and attempts at control or retaliation. Grugan (2018) examined how offenders explained inflicting cruelty on companion animals according to newspaper articles. Differences were identified between offender types, with violent offenders often being triggered by the presence or behaviour of other people’s animals, or by interpersonal disputes, whereas passive cruelty was explained by the denial of responsibility, misunderstanding the legal requirements regarding the animals, or a lack of resources. In addition, the psychopathology of animal cruelty may affect the rationalisations and depends upon the offence type: whereas animal hoarding is recognised as a syndrome in itself (e.g. Patronek 1999), intentional animal abuse is associated with antisocial behaviour and traits such as callousness and low empathy levels (e.g. Alleyne & Parfitt 2019; Diemer *et al.* 2024). To our knowledge, there is no research that would comprehend and compare the rationalisations of all offender groups.

Recently, Kaptein and van Helvoort (2019) suggested a model that includes all neutralisation techniques previously described in the literature and distinguishes four main categories based on the function of the technique in denying either the deviant behaviour or the responsibility of the actor: (1) distorting the facts; (2) negating the norms; (3) blaming the circumstances; and (4) hiding behind oneself. According to Kaptein and van Helvoort, it may be beneficial for the respondent to apply the techniques as low in the order of categories as possible, which allows them to better avoid confessing and offers them the possibility to utilise additional neutralisation techniques when necessary. This model may be even better than the prior classifications for assessing the offender’s own perception of their action. However, it has not yet been applied to the study of animal welfare offenders’ neutralisation techniques.

In this study, our aims were to: (i) identify the neutralisation techniques typically utilised in court proceedings by those charged with offences against production animals, or companion animals, violent offences, and animal hoarding offences; and (ii) explore whether using different neutralisation techniques was associated with the convictions. Information on the offender profiles and the rationalisations for offending is needed to develop animal welfare control and to prevent crimes against animals.

## Materials and methods

Our raw data comprised 1,443 judgements concerning animal welfare offences in mainland Finland between 1 January 2011 and 20 May 2021. Judgements were requested from 19 Finnish District Courts based on lists provided by the Finnish Legal Register Centre. In some judgements, two or more defendants were convicted, and every defendant was considered as a separate case. Overall, 1,628 individual defendants were identified, of whom 74 were accused in two criminal proceedings, ten in three and one in four proceedings. As their age and location, the features and targets of their offences, and the subsequent criminal sanctions differed from case-to-case, all judgements were included in the analysis as separate cases. Of all judgements, 948 with 1,137 defendants concerned offences mainly or exclusively against companion animals, and 480 judgements with 580 defendants concerned offences against production animals. Fifteen judgements with eighteen defendants concerned only wild animals, and hence these cases were excluded from the analysis. The final data therefore consisted of 1,717 cases altogether.

### Data collection and statistical analysis

We collected the data from the District Courts’ convictions as presented in Table 1. For the statistical analysis, we defined age quintiles (15–29 years, 30–39 years, 40–47 years, 48–56 years, 57–86 years). In addition, we divided the judgements into two groups: those concerning the offences against (1) companion animals (dogs, cats or other small animals kept as pets, or horses as the most numerous species), and (2) production animals (cattle, pigs, sheep, goats, poultry, reindeer, or fur animals as the most numerous species).

**Table 1.** Data collected from the District Courts’ convictions (n = 1,717 cases) of defendants charged with animal welfare offences or infringements in Finnish District Courts in 2011–2021.

Variable	Specific notes
**1. Background variables**	Age, gender
**2. Features of the offence**	
i. Animal species	
ii. Number of animals	Available for 1,635 cases, missing in 112 production animal cases
iii. Duration	Days
iv. Animal(s) dead	One or more animals dead due to the offence, or ordered to be killed by the authorities, yes/no
v. Active violence	e.g. hitting, kicking, shooting, strangling, or otherwise actively harming or killing an animal in an illegal manner, yes/no
vi. Passive maltreatment	e.g. keeping an animal without adequate nutrition, in dirty or otherwise inappropriate premises, or leaving a sick or injured animal without veterinary care, yes/no
vii. Animal’s owner	Defendant/other person
viii. Animal welfare inspection and administrative measures executed by an official veterinarian	Yes/no
**3. Witnesses and written evidence presented in court**	Position of the witness(es): veterinarian/other
**4. Defendant’s response**	Although it is obligatory for the court to hear the defendant before issuing a conviction, no response was recorded to the judgement in 38 cases
i. To the actions described in the charges	Full confession/partial confession/denial
ii. To the crime	Full confession/partial confession/denial
iii. Neutralisation techniques offered	Yes/no
iv. Neutralisation categories, techniques and subtechniques	Classified according to the model of Kaptein and van Helvoort (2019)
**5. Criminal sanctions imposed by the District Courts**	
i. Penalty: prison or probation	Yes/no
ii. Ban on the keeping of animals, duration of the ban	Yes/no, fixed/permanent
iii. Forfeiture of animals	Yes/no
**6. Other crimes that the defendant was charged with in the same court proceedings**	Related/non–related to the animal welfare offence, type of offence (e.g. traffic, property, violent offence)

For the further statistical analysis, we applied the same categorisation as in Valtonen *et al.*’s recent study (2023) to distinguish between the large-scale companion animal cases: we divided the cases involving companion animals into groups according to the number of animals involved (one animal, 2–14 animals, 15 or more animals) and the duration of the offence (1–3 days, 4–59 days, 60 days or longer) and applied the category of a large-scale offence: an offence that was committed against at least 15 animals *and* that either lasted at least 60 days *or* was repeated at least twice. As these cases were frequently shown to exhibit the typical features of animal hoarding, such as a higher mortality rate of the animal victims and a higher age of the offenders when compared to other offences against companion animals, we later call them ‘hoarding offences.’

Further, we divided the cases into three groups according to the type of offence: (1) passive neglect only; (2) violence only; and (3) both types of abuse. Concerning the offences against companion animals, the violent offence type and the large-scale offence type were almost entirely separate with only three overlapping cases. Instead, violent and passive offence types overlapped with each other in 74 cases. In 26 of these cases, the defendants offered neutralisations for both offences, in 21 cases only for the passive offence, and in five cases they offered neutralisations concerning only the violent offence. Thus, to analyse the neutralisations offered for violent vs non-violent offence types, we recorded each of these overlapping offences as two separate cases, violent and non-violent offence.

When classifying the neutralisations, we utilised the model presented by Kaptein and van Helvoort (2019) with four main categories. We recognised all four categories, eight neutralisation techniques and twenty subtechniques (Table 2). For the analysis, we formed groups of subtechniques that appeared very similar to each other and were difficult to distinguish as separate subtechniques. Thus, we recorded thirteen subtechniques or groups of subtechniques (henceforth subtechniques) that were clearly distinguishable and that recurred in more than 1% of the defendants’ responses (Table 2). Further, we explored the associations between the offence types and (1) neutralisation categories and (2) neutralisation subtechniques.

**Table 2.** Neutralisation categories, techniques and subtechniques identified in the responses given by defendants charged with animal welfare offences or infringements in Finnish District Courts in 2011–2021. Classified according to the model by Kaptein and van Helvoort (2019).

Neutralisation category	Neutralisation technique	Neutralisation subtechnique(s)
1. Distorting the facts		
	2. Denying the facts	6. The denial of the consequences of behaviour
		8. The denial of intentions
2. Negating the norm		
	4. Reducing norms to facts	19. The reduction to the immorality of accusers
		20. The reduction to an invalid norm
	5. Appealing to another norm	21. The appeal to higher goals
		23. The appeal to rights
		24. The appeal to good intentions
3. Blaming the circumstances		
	7. Blaming the limited options	31., 33., & 34. The limitation to one option, difficult, or unrealistic option(s)
	8. Blaming the limited role	37.–39. The limitation to having no responsibility, not being alone responsible, or others being responsible
		40. The limitation to the context being responsible
4. Hiding behind oneself		
	10. Hiding behind imperfect knowledge	46.–48. The hiding behind imperfect knowledge of the norm, situation, or desired conduct
	11. Hiding behind imperfect capabilities	53. & 55. The hiding behind imperfect self–restraint or self–perseverance
	12. Hiding behind imperfect intentions	56. The hiding behind imperfect mood

Associations between relevant background variables, offence types and the consequences and criminal sanctions were examined by pair-wise associations using Chi-squared tests and Fisher’s exact tests. The Mann-Whitney *U* test was used to make comparisons between the offences against production animals and companion animals concerning the age of the offender, number of animal victims, and duration of the offence.

We formed three logistic regression models to examine which factors best predicted the court convicting for a lesser offence than the prosecutor proposed or dismissing the charges altogether in different offence types (offences against production animals, violent offences, and hoarding offences). The analysis incorporated variables of the denial of charges, and the ten to thirteen relevant categories of neutralisation subtechniques, serving as explanatory variables for the models. The models were evaluated with Hosmer and Lemeshow tests for goodness of fit and ROC curves.

Statistical analyses were performed with IBM SPSS® Statistics for Windows Version 28.0 (IBM Corp, Armonk, NY, USA). Statistical significance was accepted at a confidence level of 95% (*P* < 0.05).

### Ethical approval

The studies involving human participants were reviewed and approved by the Ethical Review Board in the Humanities and Social and Behavioural Sciences, University of Helsinki, Finland. Written, informed consent for participation was not required for this study in accordance with national legislation and the institutional requirements. The participants were identified by their names and birth dates in the original documents, but the data were anonymised after collecting the necessary information on age, gender, and repeated offences.

## Results

### Offences against companion animals and production animals differ

Those defendants who were accused of crimes against production animals (henceforth animal farmers, although some defendants were employed by a farmer or a slaughterhouse), were more often male than those accused of crimes against companion animals. In the former group, the median age was higher (Table 3) and 10.5% (61/580) had reached the current Finnish retirement age of 65 years. For offences against production animals, the median number of animals was higher, and the animal victim was the offender’s property more often than in the offences targeted against companion animals (Table 3).

**Table 3.** Demographic variables, animal victims, offence types, veterinary involvement, and legal consequences in cases of animal welfare offences (n = 1,717 cases) against production animals and companion animals (including horses) charged in Finland in 2011–2021.

		Production animals, (n = 580 cases)	Companion animals (n = 1,137 cases)
Defendants	Male^a^ **	79.9%	54.6%
	Median age^b^ **	49 years (range 18–84)	40 years (15–86)
Animals	Median number^b^ **	50 (range 1–36,779)	2 (range 1–490)
	Defendant’s own^a^ **	95.7%	84.2%
Offence type	Violent^a^ **	10.0%	26.3%
	Hoarding	N/A	12.3%
	Median duration^b^ **	307 days (range 1–4,423)	36 days (range 1–3,338)
	Recurrent^a^ **	10.7%	4.5%
Veterinary involvement	Official veterinarian inspected animals^a^ **	94.7%	69.0%
	Veterinary evidence presented in court^a^ **	97.1%	82.4%
Consequences	Dead animals^a^ *	56.6%	48.5%
	Aggravated offence^a^ ^,^^c^ **	9.4%	4.2%
	Offender sentenced to prison/probation^a^ ^,^^d^ **	31.1%	14.7%
	Offender permanently banned from keeping animals^a,c^**	6.8%	3.1%
	Forfeiture of animals ordered by the court^a,c^**	22.2%	15.4%

a Chi-squared test

b Mann-Whitney *U* test

c of those defendants who were convicted of animal welfare offence(s) (n = 1,566).

d of those defendants who were convicted of animal welfare offence(s) with no other crimes (n = 1,363)

* *P* < 0.01

** *P* < 0.001

Of the companion animal cases, 12.3% were large-scale offences which referred to animal hoarding, and violent offences were more common. In production animal cases, the duration of the offence was longer, recurrent offences by the same defendant were more common, and one or more animals had died or had to be euthanased more often. (Table 3). An official veterinarian had performed an animal welfare inspection more often in cases concerning production animals and a veterinarian was also a witness in the court proceedings, and/or their inspection report, patient report or other statement was used as a piece of evidence more often than in cases involving companion animals (Table 3).

Of all defendants, 91.2% (1,566/1,717) were found guilty of at least one animal welfare offence. The offences concerning production animals were convicted as aggravated, and a permanent ban on the keeping of animals was imposed, and animals were forfeited more often than in companion animal cases (Table 3).

### Responses of the defendants

In 97.9% (1,681/1,717) of all cases, defendant’s response was recorded in the conviction. The responses had been provided during the preliminary investigation, and/or during the court hearing either orally or in writing. Of these defendants, 60.6% (1,084/1,681) denied having committed a crime, while 14.6% (245/1,681) confessed partially, and 24.4% (411/1,681) confessed to all charges. However, only 23.9% (401/1,681) of the defendants denied the actions that were described in the charges, while 45.5% (765/1,681) confessed to the actions partially, and 30.5% (513/1,681) confessed fully.

Of those defendants who provided a response during the preliminary investigation and/or the court hearing, 62.0% (1,043/1,681) offered one or more neutralisation(s). They used the neutralisation techniques in the following categories: ‘distorting the facts’ in 30.8% (321/1043), ‘negating the norm’ in 32.3% (337/1043), ‘blaming the circumstances’ in 65.1% (679/1043), and ‘hiding behind oneself’ in 36.1% (377/1043) of the cases.

### Offences against production animals and companion animals

Defendants who were charged with offences against production animals offered neutralisations more frequently (74.7%; 428/573) than those who were accused of offences against companion animals (55.5%; 615/1,108, *χ* ^2^_(2)_ = 59.1; *P* < 0.001). Nearly half of the animal farmers denied the facts, most frequently the consequences of their alleged behaviour (Table 4). They usually denied having harmed the animals, and often claimed that the animals were adapted to the conditions in which they were kept, or that the illegal conditions had only prevailed for a short period of time and hence the animals had not suffered. They often claimed that the authorities were immoral, hostile, dishonest, or unprofessional, and felt that the official veterinarian had carried out their inspection during the most inconvenient time of the day or year, behaved badly, lied, or even framed the farmer for the offence. They also challenged the authorities’ interpretation of the law more often than those charged with offences against companion animals (Table 4).

**Table 4.** Categories, techniques, and subtechniques of neutralisations utilised by the defendants (n = 1,043) charged with animal welfare offences against production animals and companion animals (including horses) in Finland in 2011–2021, classified according to the model by Kaptein and van Helvoort (2019).

Neutralisation category	Neutralisation technique	Neutralisation subtechnique(s)	Examples	% of the defendants		*χ* ^2^	*P*-value
				Offences against production animals	Offences against companion animals		
Distorting the facts				46.3	20.0	81.7	< 0.001
	Denying the facts	The denial of the consequences of behaviour	“No harm was caused to the animals.”	44.6	16.6	98.2	< 0.001
		The denial of intentions	“It was an accident.”	3.0	3.4	0.1	0.9
Negating the norm				44.2	24.1	46.6	< 0.001
	Reducing norms to facts	The reduction to the immorality of accusers	“The veterinarian is not familiar with the realities of animal production.”	36.7	8.3	127.4	< 0.001
		The reduction to an invalid norm	“The (authorities’ interpretation of the) law is invalid.”	14.7	7.2	15.7	< 0.001
	Appealing to another norm	The appeal to higher goals	“I was training the animal.”	0	4.4	19.3	< 0.001
		The appeal to rights	“It was self–defence.”	0	3.1	13.5	< 0.001
		The appeal to good intentions	“I was trying to help.”	3.0	3.7	0.4	0.6
Blaming the circumstances				72.2	60.2	16.1	< 0.001
	Blaming the limited options	The limitation to one option, difficult, or unrealistic option(s)	“It had rained very heavily.” / “I had no money.” / “I had to make the animal move/stop.”	27.1	16.6	16.9	<0.001
	Blaming the limited role	The limitation to having no responsibility, not being alone responsible, or others being responsible	“My spouse was responsible for feeding the animals.” / “The previous owner caused the animal’s malnutrition.”	31.3	30.7	0.04	0.9
		The limitation to the context being responsible	“I had a heavy workload.” / “I had just got divorced.” / “I had too many animals.”	45.8	24.7	50.4	< 0.001
Hiding behind oneself				33.2	38.2	2.8	0.1
	Hiding behind imperfect knowledge	The hiding behind imperfect knowledge of the norm, situation, or desired conduct	“It didn’t know the animal’s illness was serious.” / “I didn’t know my behaviour was prohibited by law.”	6.5	13.7	13.3	< 0.001
	Hiding behind imperfect capabilities	The hiding behind imperfect self–restraint or self–perseverance	“I was ill / injured / old / intoxicated.”	27.6	24.4	1.3	0.3
	Hiding behind imperfect intentions	The hiding behind imperfect mood	“I was provoked by the animal’s behaviour.”	0	3.1	13.5	< 0.001

In both groups, the defendants appealed most often to the circumstances (Table 4). They most frequently used the neutralisation technique of blaming their limited role and the subtechnique of claiming that the context was responsible; they typically described their heavy workload, a recent divorce, or other change in the family relations. Animal farmers also blamed difficulties in finding workers to provide hoof care, or to renovate animal premises, or claimed that not enough food was available for them to buy for their animals, or that the animals were genetically exceptionally slow growing and therefore appeared thin.

One in three defendants in both groups appealed to their limited role by claiming that they were not at all or only partly responsible for what had happened to the animals (Table 4). They held their spouse, neighbour, employee, or other agent responsible for leaving the animals without care, giving false directions, or otherwise causing the illegal situation. Further, animal farmers blamed their limited or unrealistic options more often than those charged with offences against companion animals (Table 4). Most of these defendants blamed the exceptional weather conditions or claimed to have financial problems. They often reported that they could not afford to buy food, bedding, or medication for the animals, or to renovate their premises, hire employees, or otherwise take care of their animals. On the other hand, some defendants claimed that their financial burden made it impossible to give up keeping animals because they were dependent upon the agricultural support that was determined by the number of animals. Nearly half of the animal farmers used the subtechnique of claiming that the context was responsible. Of these, 89.8% (176/196) appealed to a difficult situation, such as the illness or death of a family member, or a poor harvest. In addition, 20.4% (40/196) explained that they had too many animals, either because the slaughterhouse had failed to collect them in time or because the animals had reproduced surprisingly well.

Around one in three defendants utilised the neutralisation techniques in the category of hiding behind oneself (Table 4). Most of them referred to their imperfect capabilities due to illness or injury, or to their old age. Additionally, those charged with offences against companion animals appealed more often to not knowing the norm, situation, or desired conduct (Table 4).

With regard to animal farmers, according to a binary logistic regression analysis with the full or partial denial of charges, and all ten utilised neutralisation subtechniques as explanatory variables, three variables predicted that the court would either convict for a lesser offence than the prosecutor proposed or dismiss the charges altogether: the denial of charges (OR 3.19, CI 95% 1.77–5.72; *P* < 0.001), the denial of intentions (OR 3.10, CI 95% 1.03–9.29; *P* = 0.04), and the limitation of having no responsibility, not being alone responsible, or others being responsible (OR 1.86, CI 95% 1.15–3.01; *P* = 0.04), with *P* > 0.05 for all other explanatory variables. The model comparing the predictors was statistically significant (n = 580, *χ* ^2^_(1)_ = 28.5; *P* = 0.003), the area under the ROC curve was 64.7% (CI 58.5–70.9%; *P* < 0.001), and the *P*-value for the Hosmer and Lemeshow goodness of fit test was 0.4 (*χ* ^2^ = 7.0).

Concerning the offences against companion animals, a similar model with the full or partial denial of charges, and all thirteen relevant neutralisation subtechniques as explanatory variables showed that the denial of charges (OR 5.28, CI 95% 3.00–9.30; *P* = 0.02) and the denial of the consequences of behaviour (OR 1.78, CI 95% 1.09–2.92; *P* = 0.04) predicted the court convicting for a lesser offence than the prosecutor proposed or dismissing the charges, whereas the subtechnique of the hiding behind imperfect self-restraint or self-perseverance predicted the opposite, namely the conviction according to the charges (OR 0.42, CI 95% 0.22–0.8; *P* = 0.008). The model comparing the predictors was statistically significant (n = 1,108, *χ* ^2^_(1)_ = 85.8; *P* < 0.001), the area under the ROC curve was 69.2% (CI 65.4–73.1%; *P* < 0.001), and the *P*-value for the Hosmer and Lemeshow goodness of fit test was 0.7 (*χ* ^2^ = 3.7).

### Violent offences

When charged with a violent offence type, only 49.6% (174/351) of the defendants (henceforth violent offenders) who responded to the charges offered neutralisations, whereas 63.8% (894/1,402) did so when charged with a passive type of offence (*χ* ^2^_(1)_ = 23.8; *P* < 0.001). They used the techniques in the categories of distorting the facts and negating the norm more often than the other defendants and blamed the circumstances less frequently (Table 5).

**Table 5.** Categories, techniques, and subtechniques of neutralisations utilised by the defendants (n = 1,068) charged with violent and non-violent animal welfare offences in Finland in 2011–2021, classified according to the model by Kaptein and van Helvoort (2019).

Neutralisation category	Neutralisation technique	Neutralisation subtechnique(s)	Examples (violent offences)	% of the defendants		*χ* ^2^	*P*-value
				Violent offences	Non-violent offences		
Distorting the facts				41.4	29.3	9.9	0.002
	Denying the facts	The denial of the consequences of behaviour	“The animal didn’t suffer, it died immediately.”	33.3	27.5	2.4	0.1
		The denial of intentions	“I tried to scare the animal, but accidentally shot it.”	8.0	2.7	12.2	0.001
Negating the norm				39.7	30.4	5.7	0.02
	Reducing norms to facts	The reduction to the immorality of accusers	“Immoral / hostile authorities persecuted me.”	1.7	23.0	42.1	< 0.001
		The reduction to an invalid norm	“The (authorities’ interpretation of the) law is invalid.”	8.0	10.6	1.1	0.3
	Appealing to another norm	The appeal to higher goals	“I was training the dog / horse / cat.”	15.5	0	142.3	< 0.001
		The appeal to rights	“It was self–defence.”	10.9	0	99.4	< 0.001
		The appeal to good intentions	“I ended the animal’s suffering.” / “It was a religious slaughter.”	8.6	2.8	13.7	< 0.001
Blaming the circumstances				36.8	70.1	70.9	< 0.001
	Blaming the limited options	The limitation to one option, difficult, or unrealistic option(s)	“I had to load the horse onto a truck to transport it.” / “I tried to control the dog.”	21.3	20.9	0.01	0.9
	Blaming the limited role	The limitation to having no responsibility, not being alone responsible, or others being responsible	“I didn’t do it alone.” / “The animal caused its own injuries.”	12.6	34.1	31.6	< 0.001
		The limitation to the context being responsible	“I was so stressed.”	8.0	37.8	58.4	< 0.001
Hiding behind oneself				35.1	35.7	0.03	0.9
	Hiding behind imperfect knowledge	The hiding behind imperfect knowledge of the norm, situation, or desired conduct	“I didn’t know drowning puppies was prohibited by law.”	8.6	11.1	0.9	0.4
	Hiding behind imperfect capabilities	The hiding behind imperfect self–restraint or self–perseverance	“I was mentally ill.” / “I was intoxicated.” / “I have no memory of the situation.”	17.8	26.7	6.1	0.02
	Hiding behind imperfect intentions	The hiding behind imperfect mood	“I was provoked by the animal’s behaviour.”	10.9	0.0	99.4	< 0.001

The violent offenders appealed to accidents more often than the other defendants, for example by claiming that they had shot or dropped an animal by accident. In addition, they used the subtechnique of appealing to good intentions more often than the other defendants (Table 5), for example, by claiming that when killing an animal in an illegal manner, they had attempted to end the suffering of the animal, or to help a friend.

Within the technique of appealing to another norm, the violent offenders utilised two subtechniques that were specific only to their group. First, some of them justified their actions by explaining that they had been training an animal that behaved in an unwanted manner. In some cases, the training was harsh enough to cause the death of a cat or a dog. Second, some violent offenders appealed to their right to defend themselves, other people, or their property against an attacking animal, typically a dog (Table 5). Unlike other defendants, some of the violent offenders utilised the subtechnique of hiding behind an imperfect mood by appealing to the animal’s provocative behaviour (Table 5). These defendants explained that they had become upset or outraged, for example when a cat jumped on the table, or a dog barked or disobeyed. Overall, 48.9% (85/174) of the violent offenders appealed in some way to the animal’s behaviour, whereas only 11.0% (98/894) of the defendants did so when accused of passive maltreatment of an animal (*χ* ^2^_(1)_ = 147.3; *P* < 0.001).

According to a binary logistic regression analysis with the full or partial denial of the charges, and all thirteen utilised neutralisation subtechniques as explanatory variables, only the denial of the charges predicted the court convicting for a lesser offence than the prosecutor proposed or dismissing the charges (OR 3.19, CI 95% 1.77–5.72; *P* < 0.001), with *P* > 0.05 for all neutralisation subtechniques as explanatory variables. Additionally, despite the defendant partially or fully admitting to the crime and/or the action, the charges were dismissed more often in cases of violent offences (6.8%; 24/354) than in other cases (2.7%; 38/1,424, *χ* ^2^_(1)_ = 14.2; *P* < 0.001).

### Animal hoarding offences

The animal hoarders offered neutralisations more frequently (75.6%; 102/135) than other defendants who were charged with crimes against companion animals (52.7%; 513/973, *χ* ^2^_(1)_ = 25.0; *P* < 0.001). Most of the defendants accused of animal hoarding offences used the neutralisation techniques in the category of blaming the circumstances, and most frequently the technique of blaming their limited role (Table 6). Of the defendants denying their responsibility, all claimed that another person was either fully or partly responsible for the illegal situation. Of those who used the subtechnique of claiming that the context was responsible, 78.9% (30/38) appealed to a difficult situation, such as work overload, or a divorce, and 44.7% (17/38) explained that they had too many animals. The animal hoarders claimed significantly more often than the other defendants who were charged with crimes against companion animals that the authorities had been hostile or otherwise unprofessional or had interpreted or implemented the law in an incorrect manner (Table 6).

**Table 6.** Categories, techniques, and subtechniques of neutralisations utilised by the defendants (n = 615) charged with animal welfare offences with features of animal hoarding (hoarding offences) compared to defendants charged with other animal welfare offences against companion animals in Finland in 2011–2021, classified according to the model by Kaptein and van Helvoort (2019).

Neutralisation category	Neutralisation technique	Neutralisation subtechnique(s)	Examples (hoarding offences)	% of the defendants		*χ* ^2^	*P*-value
				Hoarding offences	Other offences		
Distorting the facts				19.6	20.1	0.01	1.0
	Denying the facts	The denial of the consequences of behaviour	“The animals were used to those conditions.” / “I would never harm an animal.”	19.6	16.0	0.8	0.4
		The denial of intentions	“It was an accident.”	1.0	3.9	2.2	0.2
Negating the norm				27.5	23.4	0.8	0.4
	Reducing norms to facts	The reduction to the immorality of accusers	“The authorities kept nagging about irrelevant details.” / “The photographs are staged.”	16.7	6.6	11.3	0.002
		The reduction to an invalid norm	“The (authorities’ interpretation of the) law is invalid.”	12.7	6.0	5.8	0.02
	Appealing to another norm	The appeal to higher goals	NA	0.0	5.3	5.6	0.03
		The appeal to rights	NA	0.0	3.7	3.9	0.06
		The appeal to good intentions	“I saved the animals from their previous owners.” / “I just fed the cats that came into my yard.”	4.9	3.5	0.5	0.6
Blaming the circumstances				74.5	57.3	10.5	0.001
	Blaming the limited options	The limitation to one option, difficult, or unrealistic option(s)	“It had rained very heavily.” / “I had no money.”	15.7	16.8	0.07	0.9
	Blaming the limited role	The limitation to having no responsibility, not being alone responsible, or others being responsible	“My spouse was responsible for feeding the animals.” / “The previous owner caused the animal’s malnutrition.”	42.2	28.5	7.5	0.007
		The limitation to the context being responsible	“Huge workload.” / “I had just got divorced.” / “I could not sell as many puppies as I had planned.”	37.3	22.2	10.3	0.002
Hiding behind oneself				28.4	40.2	5.0	0.03
	Hiding behind imperfect knowledge	The hiding behind imperfect knowledge of the norm, situation, or desired conduct	“I didn’t know the animal’s illness was serious.” / “I didn’t know it was prohibited by law.”	7.8	14.8	3.5	0.08
	Hiding behind imperfect capabilities	The hiding behind imperfect self–restraint or self–perseverance	“I was ill / injured.”	20.6	25.1	1.0	0.4
	Hiding behind imperfect intentions	The hiding behind imperfect mood	NA	0.0	3.7	3.9	0.05

According to a binary logistic regression analysis with the full or partial denial of charges, and all ten utilised neutralisation subtechniques as explanatory variables, none of the variables predicted the court convicting for a lesser offence than the prosecutor proposed or dismissing the charges (*P* > 0.05 for all explanatory variables).

## Discussion

We found that there were differences in the neutralisation techniques used by those accused of crimes against production animals and those accused of crimes against companion animals. Defendants accused of crimes against production animals or large-scale offences against companion animals denied the charges and offered neutralisations more frequently than other defendants. In contrast, those accused of crimes against companion animals in general or violent crimes against animals were less likely to respond to the charges and offered fewer explanations for their behaviour.

Although animal farmers represented only one-third of those charged with animal welfare offences, their crimes were more serious and involved larger numbers of animals, amounting to nearly 14 times more than the crimes against companion animals. The crimes against production animals led to the animal victims’ death or euthanasia more frequently, lasted longer, were more often judged as aggravated, and offenders were sentenced to prison or probation more often when the victims were production animals. Moreover, the demographics differed between the two offender groups: those charged with offences against production animals were older and more often male than those accused of crimes against companion animals. Our results are in line with Morton et al’s (2018) findings concerning the harsher penalties and higher number of animal victims in production animal cases in Australia.

Of the animal farmers, two-thirds denied having committed a crime and three out of four offered neutralisations for their behaviour, whereas less than 60% of the other defendants did so. According to the responses that were recorded in the convictions, the majority of animal farmers neutralised their behaviour with various kinds of challenging circumstances, ranging from financial problems and exceptional weather conditions to changes in family relations and having too many animals. One-third of the explanations concerned questions of responsibility: the defendants claimed that another person was either partly or fully responsible for the illegal situation. Furthermore, nearly half of the defendants denied having harmed the animals, and more than one in four appealed to physical or mental illness. Aligning the results of Väärikkälä *et al.* (2018), many blamed the authorities for being hostile or otherwise unprofessional, or even for framing them for the crime, or questioned the law or the authorities’ interpretation of it. Our results are in line also with the findings of Andrade and Anneberg (2014) in Denmark and Devitt *et al.* (2015) in the Republic of Ireland: when they interviewed farmers accused of neglecting animals, the farmers repeated narratives of financial difficulties, excessive workload, unprofessional authorities, and being in possession of too many animals relative to the resources available. The interviewees also negated the norms: some of them did not approve of the changing standards regarding animal welfare or failed to understand the legal requirements concerning their work. However, some of them referred to a sense of pride or ‘being a man’ when explaining why they did not seek help to save their suffering animals. This explanation was not detected in our study.

Financial problems were a common explanation among all defendants. In terms of animal production, they were of a multifaceted nature: on one hand, farmers claimed that they could not afford to take care of their animals, but on the other, some of them pointed out that they could not afford to give up the animal production or reduce it because they were dependent upon the agricultural support that was paid according to the number of animals. We argue that the agricultural support systems should be critically reviewed from this perspective and, if necessary, modified so that keeping too many or any suffering animals would never be profitable for the farmer. Furthermore, we agree with Andrade and Anneberg (2014), Devitt *et al.* (2015), and Väärikkälä *et al.* (2020), that an early and holistic approach to animal welfare problems is essential to better prevent crimes against production animals. As Kelly *et al.* (2011, 2013) have shown, certain indicators from register data can be used to roughly identify farms with animal welfare problems, but this is mainly in the chronic problems. In situations where animal welfare is acutely compromised, threshold for seeking and receiving help should be low. Moreover, as in the study by Devitt *et al.* (2015), some farmers in our data also cited age-related health problems or simply old age as an explanation for their inability to care for their animals. As 11% of the defendants had reached the Finnish retirement age of 65 years and the oldest defendants were over 85 at the time of committing a crime, our results raise the question of whether animal farmers are able to retire in time and receive adequate support at this stage of life.

When charged with a violent offence, the defendants admitted both the alleged behaviour and the criminal charges more often than in the cases of passive maltreatment of animals. Moreover, they offered explanations for their behaviour less frequently than the other defendants. Of those violent offenders who did neutralise their behaviour, nearly half appealed to the behaviour of the animal victim, e.g., claiming to have been provoked by it, or reporting that they had defended themselves, others, or their property against the animal, or had tried to otherwise control or train it. Further, one-third neutralised their behaviour by claiming that the animal was not hurt or had died without suffering, and nearly one in five explained that they were ill or intoxicated when committing the offence and/or had no memories of the situation. The motive of controlling an animal or being provoked by its behaviour has also been reported in previous studies (Hensley & Tallichet 2005; Newberry 2018; van Wijk *et al.* 2018). Moreover, the neutralisations that the defendants offered for their violent behaviour resembled those utilised by other violent perpetrators: blaming the victim, stating that the victim needs to obey and be punished, denying their responsibility as the victim provoked the violence (Cavanagh *et al.* 2001; Henning & Holdford 2006; Vignansky & Timor 2017), and appealing to intoxication, amnesia, or accidentally hurting the victim (Cavanagh *et al.* 2001). Thus, our results suggest the similarities and connection between domestic violence and animal abuse well demonstrated in research (e.g. Campbell *et al.* 2021; Fitzgerald *et al.* 2022).

We did not find certain motives that have been reported in previous studies, such as the motive of harming an animal for fun (Hensley & Tallichet 2005; Newberry 2018), or in order to impress or intimidate other people (Hensley & Tallichet 2005). Also, the ‘displacement of aggression’, with the person venting their frustration on an animal when being unable to retaliate against their original provoker (Pedersen *et al.* 2000), was described by Grugan *et al.* (2018) as an explanation for violence against a companion animal. These motives are not mentioned in van Wijk *et al*.’s study (2018) analysing data from police registers. We suggest that although these motives may be common, expressing them would not appear to be a plausible explanation in criminal proceedings and would therefore not be offered to the police or the courts. However, together with our recent finding of violent animal welfare offenders being regularly charged with violent offences, invasion of domestic premises, menace, or weapons offences (Valtonen *et al.* 2023), our results support the concern regarding high recidivism (e.g. Arluke *et al.* 1999; Reid & Alleyne 2024) among animal abusers, as well as the connection between animal cruelty and other forms of violence (Diemer *et al.* 2024).

Animal hoarders and animal farmers offered neutralisations more often and in larger numbers than the other defendants. Most of them used the neutralisation techniques in the category of blaming the circumstances. However, within this category, animal hoarders most often either fully or partly denied their responsibility for taking care of the animals, whereas animal farmers more typically blamed a challenging context of their workload or family problems. As in the case of animal farmers, animal hoarders also appealed to their animals being surprisingly reproductive or explained that they had not been able to sell the young animals according to their plans. Animal hoarders also claimed that the authorities had been hostile or otherwise unprofessional or had interpreted or implemented the law in an incorrect manner more often than other defendants who were charged with crimes against companion animals. Unplanned or planned breeding was a frequent explanation among animal hoarders also in the US (Arluke *et al.* 2002; Vaca-Guzman & Arluke 2005) and in Australia (Joffe *et al.* 2014; Ockenden *et al.* 2014; Elliott *et al.* 2019). Further, the hoarders’ experience of being mistreated by authorities, or even the victim of a conspiracy, has also been reported in the US (Vaca-Guzman & Arluke 2005). Vaca-Guzman and Arluke (2005) describe the ‘Good Samaritan’ justification – claiming to have saved animals from death – used by animal hoarders in the US, and according to Ockenden *et al.* (2014) and Elliott *et al.* (2019), rescuing homeless animals was a common way of acquiring large numbers of companion animals also in Australia. In our material, this neutralisation technique was not frequent, although some defendants claimed to have been feeding or otherwise taking care of homeless cats.

We analysed the associations between different neutralisation subtechniques and the court convicting for a lesser offence than the prosecutor proposed or dismissing the charges in different offender groups. The full or partial denial of charges predicted a positive outcome for the defendant in both production animal and companion animal cases. In addition, the neutralisation subtechniques of the denial of intention, such as appealing to human error or an accident, and the denial of full responsibility were associated with a positive outcome for the defendants in production animal cases. Respectively, the denial of the consequences, such as claiming that the alleged animal victims did not experience pain, predicted the court convicting for a lesser offence or dismissing the charges in companion animal cases. In contrast, the subtechnique of hiding behind imperfect self-restraint or self-perseverance, for example by claiming to have been sick or intoxicated, predicted the conviction according to the charges in companion animal cases. In violent offences, the full or partial denial of charges but none of the neutralisation subtechniques predicted the court convicting for a lesser offence or dismissing the charges, whereas in hoarding offences, neither the denial of charges nor any of the neutralisation subtechniques were associated with the outcome.

According to Kaptein and van Helvoort (2019), the lower the category of the utilised neutralisation technique, the better it allows the defendant to distance themselves from the alleged crime and leaves more room for using additional or optional neutralisation techniques in higher categories when necessary. Our results may support this theory as the two subtechniques in the first and one in the third category predicted the outcome of the court convicting for a lesser offence than the prosecutor proposed or dismissing the charges, and one subtechnique in the fourth category predicted the defendant being convicted according to the charges. However, these results should be interpreted with caution. Firstly, the responses may not predict the conviction in the same way for different offence types: although the denial of charges generally predicted the court convicting for a lesser offence or dismissing the charges, the charges of a violent offence were dismissed more often than the other charges even despite the defendant’s partial or full confession. In some cases, the judges rationalised their decisions. For example, one of them declared that as he himself had once “instinctively” kicked a strange dog sitting outside of a shop, he had to dismiss the charges of an animal welfare offence despite the defendant fully confessing to a violent offence that had resulted in the paralysis and euthanasia of a small dog. On the other hand, another judge stated that as a crime, a violent offence against a defenceless animal is similar to an assault against a person. These examples underline the need for more coherent assessment of the suffering experienced by the animal victims.

Secondly, to further analyse the causal relations between the responses of the defendants and the resulting convictions, some additional variables need to be considered, with the severity of the alleged crime being the most important. As shown in prior research, assessing the severity and intensity of the suffering of animals is a challenging task for veterinarians (Baumgaertner *et al.* 2016; Luna Fernandez *et al.* 2016), police (Lorászkó *et al.* 2023), prosecutors, and judges (Koskela *et al.* 2019; Valtonen *et al.* 2023). There are tools for assessing acute pain and discomfort, for example by interpreting the facial expressions and other behaviour of the animal (e.g. Weary *et al.* 2006), but the courts often need to assess alleged chronic suffering, its duration, intensity, and effects on animals that have not been thoroughly examined or even seen by a veterinarian. The circumstances in homes and farms may be incompletely documented and the actions of defendants difficult to verify. Furthermore, the fact that legislation allows very different practices depending upon the species makes the outcomes of criminal procedures difficult to compare. Despite these challenges, we suggest that tools will be developed for the legal assessment of different forms of suffering among all commonly kept animal species to assist and improve fair and efficient criminal procedures.

### Animal welfare implications

The differences between the types of animal welfare offences, offender types, and their underlying motivations for the offences should be considered when developing animal welfare control, agricultural support systems, and crime prevention. We suggest that timely and effective interventions in co-operation between animal welfare control, social work and healthcare are needed to reduce animal hoarding as well as to support farmers before their health or financial issues compromise the animal welfare. Violence against animals should be recognised as a form of domestic violence to ensure the safety of both humans and non-humans and to prevent further victimisation. More advanced procedures for objective assessment of the pain and suffering of animal victims in criminal procedures are needed.

## Conclusion

The offences against production animals were considered more severe in criminal procedures than those committed against companion animals. The neutralisation techniques utilised by the defendants varied between offence types. Our results confirm the observation that farmers often perceive animal welfare authorities negatively. Violent crimes against animals are often explained by the animal victim’s behaviour, resembling the neutralisation techniques used by other violent offenders.
